# Evaluation of commercial diets on fecal consistency and defecation frequency in rhesus macaques (Macaca mulatta) with chronic intermittent idiopathic diarrhea

**DOI:** 10.1186/s42826-025-00246-6

**Published:** 2025-05-20

**Authors:** Annemiek Maaskant, Niels R. Blees, Antoine Smits, Ronald J. Corbee, Jaco Bakker, Jan A. M. Langermans, Edmond J. Remarque

**Affiliations:** 1 Biomedical Primate Research Centre, Lange Kleiweg 161, Rijswijk, 2288 GJ Netherlands; 2 Department Population Health Sciences, Animals in Science and Society, Faculty of Veterinary Medicine, Utrecht University, Yalelaan 2, Utrecht, 3584 CM Netherlands; 3 Department of Clinical Sciences, Faculty of Veterinary Medicine, Utrecht University, Utrecht, 3584 CM Netherlands

**Keywords:** Monkeys, Fiber, Lactose, Waltham Faeces Score, Stool consistency, Defecation frequency, Welfare, Nutrition, SFCAs

## Abstract

**Background:**

Recurrent diarrhea is common health and welfare problem in captive rhesus macaques (*Macaca mulatta*). Aside from infectious causes, dietary factors have been implicated in diarrhea. Therefore, the main objective of this study was to evaluate commercially available pelleted diets in rhesus macaques with chronic intermittent idiopathic diarrhea. The main differences between these diets were lactose and fiber content. A randomized cross-over diet study was conducted to investigate the influence of each diet on fecal consistency and defecation frequency as indicators of diarrhea. Nine animals with chronic intermittent diarrhea and four controls were included. Each diet was fed for approximately three months, with a similar wash-out period after each diet cycle. The fecal consistency was graded using the Waltham Faeces Scoring system, with a cutoff score of > 3.5 indicating diarrhea. Both groups and diets were compared by both mixed and fixed effect models.

**Results:**

Descriptive data showed that the mean fecal consistency score was highest in the diarrhea group in the standard diet at 3.71 ± 0.456 whereas the lowest mean fecal consistency scores were observed for lactose-free and high fiber diet in both diarrhea (3.25 ± 0.423) and control group (3.04 ± 0.346). A significant improvement of the fecal consistency score was detected in the diarrhea group when fed lactose-free diets (-0.41(-0.65 – -0.16, *P* < 0.01) and -0.47(-0.68 –—0.25, *P* < 0.0002), respectively). Lactose-free and high fiber content showed the best outcome regarding improvement of the fecal consistency score -0.47(CL -0.68—- 0.25, *P* < 0.0002). Defecation frequency increased in both groups with 1.21(CL 0.65 – 1.78, *P* < 0.00001) per observation day when fed a lactose-free, high-fiber diet.

**Conclusions:**

Lactose-free and high-fiber showed overall the best improvement of the fecal consistency in animals with diarrhea. Switching to commercially available lactose-free diets may decrease diarrhea incidence in rhesus macaques suffering from chronic intermittent idiopathic diarrhea. Nevertheless, additional nutritional research is warranted and establishing optimal nutritional requirements for captive macaques will add to our ability to understand and improve dietary interventions.

**Supplementary Information:**

The online version contains supplementary material available at 10.1186/s42826-025-00246-6.

## Background

Diarrhea is a widespread health issue in captive rhesus macaques (*Macaca mulatta*). Annual diarrhea incidence of animals requiring veterinary treatment has been described to be up to 39%, with a mortality rate of 21–33% [1]. The exact etiology of diarrhea in rhesus macaques remains unclear but is likely to be multifactorial and diverse as many risk factors can be involved in causing diarrhea [1–8]. Dehydration, electrolyte disbalance, and weight loss are common sequelae of diarrhea and are challenging to manage when the animals are housed in large social groups [1, 4, 8, 9]. The prevalence of diarrhea is not restricted by age groups resulting in the loss of animals, breeding potential, loss of genetic diversity of the colony, and more importantly, a decreased welfare of these animals [1, 10].

Stool frequency and consistency is related to dietary composition in animals and humans e.g., in humans, plant-based diets have been associated with a higher bowel movement frequency as well as softer stools and the intake of fibres is described to influence the stool bulk by its water-holding capacity, stimulation of bacterial proliferation, decreased transit time, and increased gas production [11]. Diarrhea is usually defined as an increase in frequency, fluidity or volume of feces. Dietary factors have also been implicated in the occurrence of diarrhea in nonhuman primates (NHP) [2, 4–7]. Malnutrition of protein, zinc, and vitamins such as niacin, retinol, and folate, have been described to cause diarrhea in rhesus macaques [12–14]. In addition, consumption of high-fat diets can result in malabsorption and protein-losing enteropathy [15]. Food intolerances such as gluten sensitivity or lactose intolerance have also been reported [7, 16–20]. Although chronic diarrhea significantly affects animal welfare in captive macaques, very few studies have primarily focused on the influence of diet on fecal consistency in NHP.

Typically, standard research and zoo diets for macaques consist of commercially available pelleted food supplemented with of fresh fruit and/or vegetables. These diets tend to be low in fiber and high in easily digestible carbohydrates [21, 22]. In contrast to the captive diets, natural diets are high in fiber and low in fat [23, 24]. Ingredients, content, and production processes of commercial diets vary between brands. Yet, these factors could be of great influence on gastrointestinal health. Compared to other mammals such as companion animals and livestock, food trials for captive wildlife are sparse or absent. Primates are unique among mammals in having diverse digestive tract anatomy within their order [25]. With over 500 primate species (IUCN-https://www.iucnredlist.org) developing practical and scientific nutritional knowledge for each species and life stage is challenging.

At the Biomedical Primate Research Centre (BPRC, Rijswijk, The Netherlands) the same brand and formula of primate pellets were fed for decades to their NHPs. Similar to others, diarrhea is a common problem among rhesus macaques at the BPRC [1, 26–29]. Therefore, a randomized cross-over diet study was conducted to investigate whether different pelleted diets would influence fecal consistency and defecation frequency as indicators of diarrhea incidence.

## Methods

### Animals, husbandry, and housing

As previously described in Maaskant et al. [19], 14 captive-bred rhesus macaques were included in this study during 2020–2022 (Table 1). All animals were of Indian origin and bred and raised at the BPRC in several naturalistic multi-generational family groups. Ten animals had a history of chronic intermittent diarrhea that was unresponsive to conventional veterinary treatments, and infectious causes were excluded. In addition, four macaques without a history of gastrointestinal symptoms were included as controls. Both the control and diarrhea groups had not received antimicrobial treatment at least 30 days before the start of the study. The humane endpoint of this study was established when diarrhea symptoms necessitated veterinary intervention. As such, one animal with diarrhea met the humane endpoint early in the study. With only one completed diet cycle, this animal was excluded from further analysis. The remaining macaques with diarrhea (*n* = 9) were housed in three pairs and one group of three animals; the controls were housed in two pairs.

**Table 1:** Animal Characteristic

Enclosure	ID	Age (yrs)	Gender	BW (kg)	Group	Diet sequence
1	RM 1	4	M	8.1	Diarrhea	LFLF, STAN, LCMF-hy, STAN, STAN, STAN, LFHF, STAN, LCMF-ex
	RM2	4	M	8.3	Diarrhea	LFLF, STAN, LCMF-hy, STAN, STAN, STAN, LFHF, STAN, LCMF-ex
2	RM3	5	F	7.9	Diarrhea	LFHF, STAN, LCMF-ex, STAN, STAN, STAN, LCMF-hy, STAN, LFLF
	RM4	7	F	8.5	Diarrhea	LFHF, STAN, LCMF-ex, STAN, STAN, STAN, LCMF-hy, STAN, LFLF
3	RM5	5	M	10.8	Diarrhea	LCMF-hy, STAN, LFLF, STAN, LCMF-ex, STAN, STAN, STAN, LFHF
	RM6	5	M	10.0	Diarrhea	LCMF-hy, STAN, LFLF, STAN, LCMF-ex, STAN, STAN, STAN, LFHF
	RM7	5	M	9.5	Diarrhea	LCMF-hy, STAN, LFLF, STAN, LCMF-ex, STAN, STAN, STAN, LFHF
4	RM8	3	M	7.4	Control	LCMF-ex, STAN, LFLF, STAN, STAN, STAN, LCMF-hy, STAN, LFHF
	RM9	3	M	6.0	Control	LCMF-ex, STAN, LFLF, STAN, STAN, STAN, LCMF-hy, STAN, LFHF
5	RM10	5	M	10.1	Control	LCMF-ex, STAN, LFLF, STAN, STAN, STAN, LCMF-hy, STAN, LFHF
	RM11	5	M	10.7	Control	LCMF-ex, STAN, LFLF, STAN, STAN, STAN, LCMF-hy, STAN, LFHF
6	RM12	7	M	11.1	Diarrhea	LCMF-hy, STAN, LFHF, STAN, LFLF, STAN, STAN, STAN, LCMF-ex
	RM13	7	M	12.4	Diarrhea	LCMF-hy, STAN, LFHF, STAN, LFLF, STAN, STAN, STAN, LCMF-ex

Animal characteristics including subjects’ social grouping, gender, age (years) at the start of the study, bodyweight (kg), and the diet sequence. The standard BPRC diet (STAN) was used as a washout period and was also included in the experimental rotation. The other diets were classified as lactose-containing medium fiber (LCMF), lactose-free low fiber (LFLF), and lactose-free high fiber (LFHF). LCMF was manufactured in two ways; a high-pressure and high-temperature extrusion process (LCMF-ex) and a conventional extrusion process (LCMF-hy)

The animal enclosures were divided into an indoor and outdoor compartment; the indoor floor was provided with wood fiber bedding (Lignocel3-4, JRS, Rosenberg, Germany) and the outdoor compartment consisted of sand bedding. The wood fiber bedding of the indoor compartments was replaced weekly; one week without additional cleaning procedures and one week with high-pressure water cleaning including disinfection (Anistel Surface disinfectant, Tristel Solutions Limited, Cambridgeshire, United Kingdom) as described elsewhere [30]. Standard environmental enrichment in these enclosures consisted of several climbing structures, beams, fire hoses, and sitting platforms. The indoor temperature was set at 18 °C, with a 12-h light:dark cycle.

The animals were fed ~ 150 g commercial monkey pellets (Ssniff, Soest, Germany) per day supplemented with limited amounts of fruit, vegetables (~ 150 g), or grain mixtures (~ 20 g). The pellets were provided once daily in the morning in multiple feeding trays for each enclosure. The supplemental food items i.e., fruits, vegetables or grains, were similar among each group and were provided once daily in the afternoon. Actual food intake per individual was not recorded. The five most fed fruits and vegetables were banana, maize silage, apple, cabbage, and endive. Banana and maize silage were given once a week, the frequency of the other items depended on season and availability. Water was available ad libitum, provided by automatic water dispensers.

Animal caretakers observed the animals at least twice daily for injuries and illness; abnormalities were reported to veterinarians. Individual electronic health records were kept for each animal.

### Diets

Five commercially available diets, including the BPRC standard diet (STAN), were selected for this study. Each diet differed in ingredients, content, or preparation (Table 2). The other four diets were classified based on lactose and fiber content: two lactose-containing medium fiber (LCMF) diets, one lactose-free low fiber (LFLF), and one lactose-free high fiber (LFHF). LCMF were both extruded diets; during this process, the raw ingredients were exposed to high pressure and high temperatures (> 100 °C). One LCMF diet was manufactured during a high-pressure and high-temperature extrusion process (LCMF-ex), while the second was manufactured with a conventional extrusion process (LCMF-hy). Full product names, as provided by the manufacturers, are shown in Table A1 (Additional file 1).

**Table 2:** Main diet composition according to the specifics published or provided by each manufacturer

Preparation	**Diet**				
	STAN	LCMF-ex	LCMF-hy	LFLF	LFHF
	Pelleted	Extrusion	Hybrid	Pelleted	Pelleted
Metabolized energy (kcal/kg)	3275	3382	3382	3418	2979
Fat (% energy)	12	12	12	13	11
Protein (% energy)	31	26	26	26	23
Carbohydrates (% energy)	57	60	60	61	67
Vitamin B12 (μg/kg)	100	24	24	100	28
Folic acid (mg/kg)	7	2	2	7	6
Vitamin A (IU/kg)	15,000	15,000	15,000	15,000	20,000
Nicotic acid (mg/kg)	102	36	36	86	21^a^
Zinc (mg/kg)	86	77	77	73	88
	%	%	%	%	%
Moisture	11.9	10.2	10.2	10.3	10^b^
Crude fat	4.3	4.6	4.6	5.0	3.5
Crude protein	25.2	22.2	22.2	22.0	17.0
N-free extractives	47	52	52	52.2	49.6
Crude fiber	4.2	4.5	4.5	3.5	14.0
NDF	11.8	n/a^c^	n/a^c^	10.2	27
ADF	6.4	n/a^c^	n/a^c^	3.8	16.4
Starch	27.6	29.8	29.8	38.8	26.7
Sugar	8.5	12.9	12.9	4.4	5.1
Ash	7.4	6.5	6.5	7.0	5.9
Lactose	3.5	3.4	3.4	-	-
Calcium	1.1	0.71	0.71	1.1	0.74
Potassium	1.25	0.98	0.98	0.59	0.81
Sodium	0.2	0.24	0.24	0.25	0.25
Phosphorus	0.8	0.51	0.51	0.8	0.6
Histidine	0.63	0.55	0.55	0.52	0.4
Isoleucine	1.08	1.0	1.0	1.01	0.7
Leucine	2.01	1.7	1.7	2.32	1.3
Methionine	0.55	1.19	1.19	0.45	0.4
Phenylalanine	1.15	1.04	1.04	1.19	0.8
Threonine	0.98	0.83	0.83	0.9	0.7
Tryptophan	0.31	0.29	0.29	0.24	0.2
Valine	1.16	1.09	1.09	1.18	0.9
Lysine	1.45	1.19	1.19	1.28	0.9

The standard BPRC diet (STAN), the other diets were classified as lactose-containing medium fiber (LCMF); manufactured by high-pressure and high-temperature extrusion process (LCMF-ex) and a conventional extrusion process (LCMF-hy); lactose-free low fiber (LFLF); and lactose-free high fiber (LFHF)

^a^Niacinamide

^b^Estimated

^c^Not available

### Study design

All animals were randomly assigned to a specific diet order (Table 1). After each dietary cycle with a duration of approximately three months, a wash-out period with STAN for a similar duration was implemented. To ensure a gradual transition, each diet transition started with 75% old diet and 25% new diet for two days, followed by 50% old and 50% new diet for another two days, and then 25% old and 75% new diet for the final two days, ending in 100% new diet by the seventh day.

The defecation frequency was recorded by counting the number of stools deposited by each individual within their indoor enclosure, with each stool additionally graded for fecal consistency. The fecal consistency of each animal was scored by using the Waltham Faeces Scoring system by two experienced neutral observers. The Waltham scale utilizes a scale of 1–5 with half numerical increments, covering a range of very firm and dry (score 1) to entire liquid stool (score 5) [31, 32]. The mean Waltham score was based on the average daily fecal scores of days 37, 51 and 65 of each dietary cycle. This sampling period allowed for a sufficient habituation of each animal to dietary change before assigning the Waltham scores. A mean daily Waltham score of > 3.5 was considered diarrhea.

To identify the feces from each animal, animals were randomly selected to voluntarily drink 20 mL diluted syrup (± 1:6) (RAAK, United Soft Drinks BV, Utrecht, NL) supplemented with nothing, with 0.3 mL blue food color (Wilton Industries, Icing color, Royal blue, Naperville, USA), or with 0.35 g non-digestible glitter (The sparkle range, Rainbow dust.co.uk, Cuerden Greenmill, UK). All animals received the food color and glitters in alternating sequences to exclude the possible influence of these identification methods. The animals were offered food color or glitters in the afternoon and the next morning the number of stools, i.e. defecation frequency, and fecal consistency was recorded.

### Statistical analyses

Statistical analyses were performed in RStudio (using R version 4.4.1; R Core Team, 2023). Data of observations days 37, 51 and 65 of each diet were used in the analysis, taken together these periods include 178 fecal scores. Fecal scores were averaged per day and throughout day 37, 51 and 65 to calculate the average daily fecal consistency score. For each analysis, the data were reported for all animals, the diarrhea group, and the control group.

The influence of diet on fecal consistency and average daily defecation frequency was evaluated using mixed-effects models. A step-up selection model was used to fit fixed factors of diet, housing combination, gender, the simultaneous presence of food color, and interactions, with random factors for the individual animal, diarrhea animals, the controls, and the order of diets. Akaike’s information criterion and likelihood-ratio tests were used to compare models. The assumptions for mixed-effects models, i.e. normality of residuals, homogeneity of variance, and independence of random effects were met for each model. *P-*values < 0.05 were considered statistically significant.

### Model summary

The model for average daily fecal scores consisted of diet and the presence of food colorant as fixed factors. The individual animal, diarrhea animals, the controls, and the order of diets were random factors. The final models for daily defecation frequency and average periodic defecation frequency contained only diet as a fixed factor and the individual animal as a random factor. An autoregressive covariance structure provided the best residual covariance structure for average daily fecal scores. For defecation frequencies, a diagonal covariance structure provided the best fit.

When including relative dietary macronutrient levels, the model for average daily fecal scores included lactose content and food colorant as fixed factors and individual animals, diarrhea animals, the controls as random factor. The models for daily defecation frequencies included presence of dietary lactose and dietary fiber levels as fixed factors. The individual animals were random factors.

## Results

One pair e.g., RM3 and RM4, showed a selective feeding behavior during the transition period to LCMF-ex. A portion of the pelleted food was left uneaten (range 60–180 g/day) on seven subsequent days, while the supplemental food was consumed well. The transition period was extended by 1 week for 25% old and 75% new. Hereafter, the new diets were consumed as normal.

### Descriptive data

Descriptive data of fecal consistency scores and defecation frequency, categorized by group and diet, are summarized in Table 3. The complete descriptive dataset is available in Table A2 and A3 (Additional file 2). The mean fecal consistency score was highest in the diarrhea group for STAN 3.71 ± 0.456. For LFHF we observed the lowest mean fecal consistency scores in both diarrhea and control groups. The maximum daily fecal scores in the control group were 4.0–4.5, even though the mean fecal scores of all diets in the control group did not exceed 3.5 (Table A2 (Additional file 2)), indicating that the animals in the control group showed incidental bouts of diarrhea.

**Table 3:** Summary statistics fecal consistency score and defecation frequency

Group	Diet	Fecal consistency	Defecation frequency
		**Mean** ± **SD**	**Mean** ± **SD**
All	STAN	3.63 ± 0.424	3.36 ± 1.367
	LCMF-ex	3.51 ± 0.401	3.61 ± 1.358
	LCMF-hy	3.54 ± 0.482	2.44 ± 1.231
	LFLF	3.32 ± 0.354	3.14 ± 1.475
	LFHF	3.20 ± 0.410	4.31 ± 1.635
Diarrhea	STAN	3.71 ± 0.456	3.48 ± 1.503
	LCMF-ex	3.55 ± 0.444	3.95 ± 1.311
	LCMF-hy	3.56 ± 0.520	2.33 ± 1.038
	LFLF	3.30 ± 0.368	3.12 ± 1.201
	LFHF	3.25 ± 0.423	4.25 ± 1.751
Control	STAN	3.43 ± 0.269	3.08 ± 0.996
	LCMF-ex	3.46 ± 0.334	3.08 ± 1.311
	LCMF-hy	3.50 ± 0.400	2.67 ± 1.614
	LFLF	3.36 ± 0.335	3.17 ± 1.992
	LFHF	3.04 ± 0.346	4.50 ± 1.309

Summary statistics of the raw data of fecal consistency score and defecation frequency, presented for all animals and divided into control and diarrhea groups, mean ± standard deviation (SD). The fecal consistency was based on the mean Waltham score and the defecation frequency the mean number of stools that were recorded during the observation days

Regarding defecation frequency, LCMF-hy showed overall the lowest mean frequency and LFHF the highest mean frequency.

### Effect of diet on average fecal consistency score

When controlling for the effect of food coloration used in all animals in all diet cycles on fecal consistency, the fixed effect analysis showed a statistically significant increased mean effect of 0.13 (CL 0.02–0.24, *P* = 0.02). An increase of 0.23 (CL 0.09 − 0.37; *P* < 0.002) was observed for the control group separately compared to a nonsignificant effect of 0.09 (CL −0.07 − 0.24; *P* < 0.300) in the diarrhea group.

**Fig. 1:**
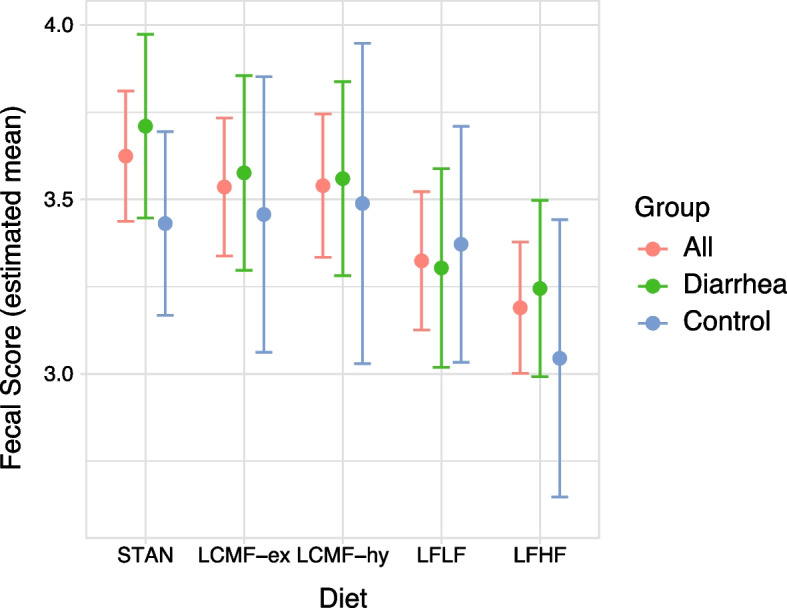
Mixed model results showing estimated mean fecal consistency scores, and whiskers indicate a 95% confidence interval for each diet, for all animals (red, left) and for the diarrhea (green, middle) and control group (blue, right) separately. In the diarrhea group, both LFLF and LFHF showed a decrease in fecal score, in the control group LFHF showed a decrease in mean score

Figure 1 shows the results of the mixed model outcomes as estimated mean fecal scores with 95% confidence intervals between the diets and the groups. In the control group, diets STAN, LCMF-ex, and LCMF-hy showed no influence of diet on fecal scores whereas LFHF showed a decrease in the estimated mean fecal score. Further analysis was performed in the fixed effect model, STAN was used as a reference diet e.g., intercept or index in mixed model coefficients. The diarrhea group showed a significant decrease in fecal scoring in LFLF and LFHF, with an estimated mean decreased fecal score of −0.41 (CL −0.65 – −0.16; *P* = 0.002) and −0.47 (CL −0.68 – −0.25; *P* < 0.0002), respectively. In the control group, compared to STAN similar fecal scores in diets LCMF-ex, LCMF-hy and LFLF were observed. LFHF showed a significant decrease in estimated mean fecal score of −0.39 (CL −0.68 – 0.09; *P* < 0.02). The estimated coefficients for diet type on fecal score from the fixed model are shown in Table 4.

**Table 4:** Results fixed factors model for fecal consistency scoring

Group	Diet	Estimate	SEM	*P* value	Lower CL	Upper CL
All	Index	3.56	0.086	**-**	3.39	3.73
	LCMF-ex	−0.09	0.092	0.34287	−0.27	0.10
	LCMF-hy	−0.08	0.097	0.38450	−0.28	0.11
	LFLF	−0.30	0.092	**0.00213**	−0.49	−0.11
	LFHF	−0.43	0.089	**0.00001**	−0.61	−0.26
Diarrhea	Index	3.67	0.118	**-**	3.43	3.90
	LCMF-ex	−0.13	0.118	0.26748	−0.38	0.11
	LCMF-hy	−0.15	0.119	0.21454	−0.39	0.09
	LFLF	−0.41	0.121	**0.00211**	−0.65	−0.16
	LFHF	−0.47	0.107	**0.00015**	−0.68	−0.25
Control	Index	3.32	0.089	**-**	3.14	3.50
	LCMF-ex	0.03	0.134	0.85017	−0.27	0.32
	LCMF-hy	0.06	0.153	0.71367	−0.28	0.39
	LFLF	−0.06	0.118	0.62394	−0.32	0.20
	LFHF	−0.39	0.135	**0.01431**	−0.68	−0.09

Results fixed factors model for fecal consistency scoring including diet, housing combination, gender, and interactions, with random factors for the individual animal and the order of diets, both lower and upper confidence intervals are presented (Upper CL and Lower CL). The index is diet A without the influence of food color or glitter. Significant *P* values are in bold

**Fig. 2:**
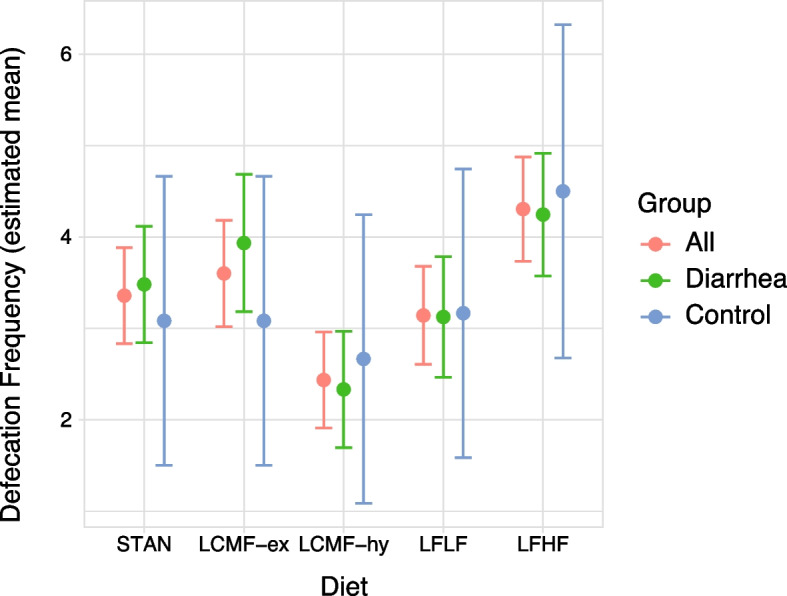
Mixed model results showing estimated mean defecation frequencies. The Whiskers indicate a 95% confidence interval for each diet, for all animals (red, left) and for the diarrhea (green, middle) and control group (blue, right) separately. LFHF showed an increase in defecation frequencies, compared to an overall decrease for LCMF-hy

Figure 2 shows the mixed model outcomes as estimated mean defecation frequency with 95% confidence intervals between the diets and the groups. Interestingly, LFHF showed the highest estimated mean defecation frequencies in all groups: 4.31 (CL 3.74–4.88), 4.25 (CL 3.57–4.92), 4.50 (CL 2.68–6.32), for all, diarrhea and control group respectively. In line with mixed model results, LFHF had significantly higher defecation frequency compared to STAN. In addition, LCMF-hy had significantly lower defecation frequencies than STAN. Table A4 (Additional file 3) shows the estimated coefficients for diet type on defecation frequency.

### Impact of macronutrient composition on fecal consistency and defecation frequency

On the macronutrient level, lactose had the most impact on the fecal consistency in the diarrhea group. When fed lactose-containing diets, the fecal consistency scores significantly increased by 0.35 (CL 0.18 − 0.51; *P* < 0.00002) compared to the control animals showing an increase of 0.23 (CL 0.02 − 0.45; *P* < 0.05).

The defecation frequency is mostly affected by high fiber content (increased defecation frequency), and unaffected by lactose content (Fig A1 (Additional file 4)). Table 5 presents the differences in defecation frequencies and the macronutrient compositions, where the index group consisted of medium fiber and lactose.

**Table 5:** Results of the fixed factor models for defecation frequency

Group	Fixed macronutrients	Value	SEM	*P*-value	Lower CL	Upper CL
All	Index	3.10	0.167		2.76	3.43
	Low fiber—lactose-free	0.05	0.271	0.86325	−0.49	0.58
	High fiber – lactose-free	1.21	0.286	**< 0.00001**	0.65	1.78
Diarrhea	Index	3.17	0.191		2.79	3.55
	Low fiber – lactose-free	−0.05	0.335	0.89183	−0.71	0.62
	High fiber – lactose-free	1.08	0.341	**0.00204**	0.40	1.75
Control	Index	2.94	0.369		2.20	3.69
	Low fiber – lactose-free	0.22	0.461	0.63225	−0.70	1.15
	High fiber—lactose-free	1.56	0.541	**0.00593**	0.47	2.64

Results of the fixed factor models for defecation frequency, index is medium fiber and lactose compared to different classes of fiber content (low and high) and absence of lactose in the diets. Significant *P* values are in bold

Compared to the index i.e., medium fiber and lactose, high fiber content and the absence of lactose significantly increased the defecation frequency in all, diarrhea and the control group, with 1.21 (CL 0.65–1.78, *P* < 0.00001), 1.08 (CL 0.04–1.75, *P* = 0.002) and 1.56 (CL 0.47–2.64, *P* = 0.0059), respectively. Low fiber content had no appreciable influence on the defecation frequency.

## Discussion

Our study identified variations in fecal consistency score and defecation frequency in captive rhesus macaques fed different commercially available pellets. On the macronutrient level, the primary differences were lactose content and dietary fiber. Lactose-free diets showed the most profound improvement in fecal consistency in macaques with intermittent idiopathic diarrhea. High fiber increased the defecation frequency but not the fecal consistency. Overall, LFLF and LFHF showed the best results in improving fecal consistency scores compared to STAN.

The lactose-free diets (LFLF and LFHF) showed a significant improvement in the fecal score compared to the other diets (STAN, LCMF-ex, and LCMF-hy), suggesting that lactose contributes to the diarrhea symptoms. A few studies describe lactose intolerance in rhesus macaques [17, 19, 20]. Hart et al. reported lactose intolerance in two sibling rhesus macaques [17]. Both infants, raised on milk formula, experienced diarrhea and slow weight gain, which resolved when transitioned to lactose-free formula. Wen et al. reported a 100% incidence of lactose intolerance in adult wild-caught rhesus macaques when they were fed with lactose-rich diets [20]. Nevertheless, the macaques demonstrated an adaptation to prolonged lactose feeding. Our study group comprised individuals of the fourth generation or beyond at the BPRC and the same lactose-containing STAN was fed for many years. Yet, our diets had relatively low lactose levels at 3.4% and 3.5% compared to 20% lactose-containing diets fed in the study by Wen et al*.* [20]. Adaptation to lactose levels in macaques at the BPRC was expected, however, our study indicates that several macaques with diarrhea remain lactose intolerant despite multigenerational captivity and lifelong exposure to dietary lactose. Microbiome data obtained from this study supported this lactose intolerance hypothesis and is described in detail elsewhere [19].

In all animals, LFHF induced the largest improvement in fecal consistency, and it also significantly increased the defecation frequency compared to the standard diet. Some dietary fibers have prebiotic properties by increasing the abundance of butyrate and other SCFA-producing bacteria [33–37]. The digestibility or fermentation efficiency of dietary fibers depends on the fermentation strategy of the animal species [38–40]. Macaques are hind-gut fermenters and the microbiome in the well-developed caecum and colon facilitates the fermentation of the dietary fiber [25, 41]. The increased defecation frequency could also be explained by the water holding and water swelling capacity of dietary fiber, which expands the fecal volume and transit time [42]. Insoluble fibers such as cellulose can increase the fecal volume/bulk and decrease intestinal transit [43]. However, Brodribb et al. [44] showed in *Macaca arctoides* that a diet with an increase fiber content to up to 20 g/day did not influence the percentage of water content in the feces. Conversely, in humans, the deprivation of dietary fiber resulted in a decrease in stool bulk and frequency [45].

The National Research Council guidelines for nutrient requirements of nonhuman primates for fiber content in total dietary dry matter of extruded diets, are NDF 10 and ADF 5 for macaque-species [12]. These guidelines are mainly based on one reference [41]. In our study, LFHF not only contained more crude fiber (14%) compared to the other diets (range 3.4–4.5%) but also consisted of a higher NDF (27%) and ADF (16.4%) level compared to the others (range NDF 10–12%; ADF 3.8–6.4%). For Diets LCMF-ex and LCMF-hy, these values were not available. The increased defecation frequency could be explained by a higher ADF and thus a higher content of cellulose [46, 47].

Protein is an essential dietary component, fundamental for maintenance, growth, and overall health. The main protein sources in our diets were unclear, the reported diet ingredients by the manufacturers were incomplete. In general, grain and grain byproducts are important sources of protein in NHP diets. In addition, STAN, LCMF-ex, and LCMF-hy included protein-rich milk byproducts such as whey and cream powder. The nutritional quality of a protein is heavily influenced by its amino acid composition, and the degree to which an essential amino acid becomes limiting depends in part on growth rate. Both dietary protein deficiency and protein excess can lead to diarrhea among other symptoms [13, 48]. At the BPRC, no other symptoms such as edema, poor musculature, skin lesions, or haircoat abnormalities were observed.

All five diets contained similar levels of protein; the crude protein content of each diet was above the estimated minimal requirement of 8% for macaques and above the proposed adequate NHP concentration of 15–22% [12]. The estimated apparent protein digestibility of food in captive rhesus macaques is 65–84% [49]. In general, the digestibility of animal protein sources is higher compared to plant protein sources [50, 51]. Although STAN, LCMF-ex, and LCMF-hy contained whey or cream and milk powder, the total amounts of these ingredients were relatively low at 3.4–3.5%. Therefore, it is unlikely that dietary protein deficiency or protein excess has contributed to the occurrence of diarrhea in the studied subjects.

Vitamins are essential for several physiological and biochemical functions in the body. Although optimal vitamin requirements for macaques are not well-defined or even incongruent, deficiency signs due to experimental deprivation are relatively well studied in these species. Depending on the specific vitamin, deficiency symptoms may include diarrhea, loss of appetite, weight loss, alopecia, and anemia. Specifically, deficiencies in niacin, folate, and retinol are described to cause diarrhea in rhesus macaques [14]. In addition, adequate dietary zinc is necessary for the maintenance of the normal plasma concentration of retinol [52]. All diets contained sufficient levels of folate, retinol, and zinc and above NRC 2003 requirements (ranges in the diets; zinc 73–88 mg; retinol 15,000–20000 IU/kg; folate 2–7 mg/kg; niacin 21–102). Deficiencies in these vitamins are therefore unlikely and can be excluded as significant factors in the etiology of diarrhea in our cohort. Overall, the differences in vitamin content between the diets were relatively small. Yet, it remains unknown if the diets contain optimum levels of vitamins for rhesus macaques.

However, some study limitation should be acknowledged. First, commercial diets were used as they could be implemented directly into our dietary management. These commercial diets also present relatively small variations in ingredient composition and micronutrient content which reduces the ability to draw definitive conclusions solely on the influence of macronutrient composition. Second, only feces present in the indoor enclosures were scored, therefore, the defecation frequency could be slightly biased. In addition, due to the intermittent character, the variability in severity of the diarrhea, and a scoring day every two weeks, the observed fecal scoring appeared lower compared to the daily subjective observations. Indicating that the diarrhea was more severe than the results indicate and thus a minor decrease in mean fecal consistency score corresponds to a more substantial clinical improvement (personal observations). Yet, the fecal scoring method was sufficient to monitor changes in fecal consistency.

The impact of unknown confounding factors such as season and environmental factors such as stress were reduced by the random assignment of the diets. In addition, digestibility and absorption of micro- and macronutrients are also influenced by multiple factors such as the nutrient content of the supplemented vegetables and fruits. Furthermore, nutritional requirements for rhesus macaques are mainly based on deficiency studies and a few studies in which wild primate diets were analyzed. Therefore, experimental dietary trials for macaques are recommended to establish optimal nutritional requirements in captivity. Increased nutritional knowledge will result in better health outcomes for rhesus macaques.

## Conclusions

LFLF and LFHF induced the best improvement of fecal consistency, possibly due to the absence of lactose in these two diets. LFHF showed the best improvement in fecal consistency and increased the defecation frequency, likely due to the relatively higher fiber content compared to LFLF. Additional nutritional research is warranted, establishing optimal nutritional requirements for captive macaques will add to our ability to understand and improve dietary interventions. Improved dietary management practices will ensure healthier and more resilient captive primate populations.

## Supplementary Information

Additional file 1.

Additional file 2.

Additional file 3.

Additional file 4.

## Data Availability

Data are available from the corresponding author upon reasonable request.

## Abbreviations

BPRC: Biomedical Primate Research Centre
LCMF-ex: Lactose Containing Mid Fiber Extruded
LCMF-hy: Lactose Containing Mid Fiber Hybrid
LFHF: Lactose Free High Fiber
LFLF: Lactose Free Low Fiber
NHP: Non-Human Primate
STAN: Standard diet, Lactose containing mid fiber
