# Supplementary material for: Evaluation of commercial diets on fecal consistency and defecation frequency in rhesus macaques (Macaca mulatta) with chronic intermittent idiopathic diarrhea
Source: Lab Anim Res. 2025 May 20;41:15. doi: 10.1186/s42826-025-00246-6 (PMC12090390; doi:10.1186/s42826-025-00246-6)
Supplement: Supplementary file 1 — Additional file 1. [file 42826_2025_246_MOESM1_ESM.pdf]

Additional file 1

Table A1. Diet coding, product names and manufacturer details.

**Diet**	**Product name**	**Manufacturer**
STAN	Primate/NHP pellets	Ssniff, Soest, Germany
LCMF-ex	Extrudate 6029	Altromin, Lage, Germany
LCMF-hy	Hybrid 6028	Altromin, Lage, Germany
LFLF	Primate/NHP vegetarian	Ssniff, Soest, Germany
LFHF	Primate PT 1 pellets	Kasper Faunafood, Woerden, the Netherlands
