# Supplementary material for: Evaluation of commercial diets on fecal consistency and defecation frequency in rhesus macaques (Macaca mulatta) with chronic intermittent idiopathic diarrhea
Source: Lab Anim Res. 2025 May 20;41:15. doi: 10.1186/s42826-025-00246-6 (PMC12090390; doi:10.1186/s42826-025-00246-6)
Supplement: Supplementary file 2 — Additional file 2. [file 42826_2025_246_MOESM2_ESM.pdf]

Additional file 2

Table A2 Summary statistics of the raw data of the fecal consistency score

Group	Diet	Count	Mean	SD	Lower CL	Upper CL	Median	P25	P75	Min	Max
All	STAN	39	3.63	0.424	3.5	3.8	3.5	3.4	3.9	3.0	5.0
	LCMF-ex	31	3.51	0.401	3.4	3.7	3.4	3.3	3.7	3.0	4.7
	LCMF-hy	39	3.54	0.482	3.4	3.7	3.5	3.3	3.8	2.5	4.9
	LFLF	37	3.32	0.354	3.2	3.4	3.3	3.0	3.5	2.5	4.0
	LFHF	32	3.20	0.410	3.1	3.3	3.1	3.0	3.4	2.3	4.5
Diarrhea	STAN	27	3.71	0.456	3.5	3.9	3.5	3.4	3.9	3.0	5.0
	LCMF-ex	19	3.55	0.444	3.3	3.8	3.4	3.3	3.8	3.0	4.7
	LCMF-hy	27	3.56	0.520	3.4	3.8	3.5	3.3	3.8	2.5	4.9
	LFLF	25	3.30	0.368	3.1	3.5	3.3	3.0	3.5	2.5	4.0
	LFHF	24	3.25	0.423	3.1	3.4	3.1	3.0	3.4	2.5	4.5
Control	STAN	12	3.43	0.269	3.3	3.6	3.5	3.3	3.5	3.0	4.0
	LCMF-ex	12	3.46	0.334	3.2	3.7	3.4	3.3	3.7	3.0	4.0
	LCMF-hy	12	3.50	0.400	3.2	3.8	3.5	3.3	3.5	3.0	4.5
	LFLF	12	3.36	0.335	3.1	3.6	3.3	3.1	3.5	3.0	4.0
	LFHF	8	3.04	0.346	2.8	3.3	3.1	3.0	3.2	2.3	3.4

Summary statistics of the raw data of the fecal consistency score, presented for all animals and divided into control and diarrhea groups, number of observations per diet, mean, standard deviation (SD), and both lower and upper confidence intervals are presented (Upper CL and Lower CL) and minimum (Min) and maximum (Max) observation.

Table A3 Summary statistics of the raw data of the defecation frequency

Group	Diet	Count	Mean	SD	Lower CL	Upper CL	Median	P25	P75	Min	Max
All	STAN	39	3.36	1.367	2.9	3.8	3	2	4	1	6
	LCMF-ex	31	3.61	1.358	3.1	4.1	4	3	4.5	1	6
	LCMF-hy	39	2.44	1.231	2.0	2.8	2	1	3	1	6
	LFLF	37	3.14	1.475	2.6	3.6	3	2	4	1	6
	LFHF	32	4.31	1.635	3.7	4.9	5	3	6	1	6
Diarrhea	STAN	27	3.48	1.503	2.9	4.1	4	2	4	1	6
	LCMF-ex	19	3.95	1.311	3.3	4.6	4	3	5	2	6

	LCMF-hy	27	2.33	1.038	1.9	2.7	2	1.5	3	1	4
	LFLF	25	3.12	1.201	2.6	3.6	3	2	4	1	6
	LFHF	24	4.25	1.751	3.5	5.0	5	2.8	6	1	6
Control	STAN	12	3.08	0.996	2.5	3.7	3	2	4	2	5
	LCMF-ex	12	3.08	1.311	2.3	3.9	3	2.8	4	1	5
	LCMF-hy	12	2.67	1.614	1.6	3.7	2.5	1	4	1	6
	LFLF	12	3.17	1.992	1.9	4.4	3	1	5	1	6
	LFHF	8	4.50	1.309	3.4	5.6	4.5	4	5.3	2	6

Summary statistics of the raw data of the defecation frequency, presented for all animals and divided into control and diarrhea groups, number of observations per diet, mean, standard deviation (SD), and both lower and upper confidence intervals are presented (Upper CL and Lower CL) and minimum (Min) and maximum (Max) observation.
