# Supplementary material for: Evaluation of commercial diets on fecal consistency and defecation frequency in rhesus macaques (Macaca mulatta) with chronic intermittent idiopathic diarrhea
Source: Lab Anim Res. 2025 May 20;41:15. doi: 10.1186/s42826-025-00246-6 (PMC12090390; doi:10.1186/s42826-025-00246-6)
Supplement: Supplementary file 3 — Additional file 3. [file 42826_2025_246_MOESM3_ESM.pdf]

Additional file 3

Table A4 Results fixed factors model for defecation frequency

Group	Fixed diet	Value	SEM	*P* value	Lower CL	Upper CL
All	Index	3.36	0.240	-	2.88	3.83
	LCMF-ex	0.24	0.331	0.4650	-0.41	0.90
	LCMF-hy	-0.92	0.310	**0.0034**	-1.54	-0.31
	LFLF	-0.22	0.314	0.4908	-0.84	0.40
	LFHF	0.95	0.327	**0.0043**	0.30	1.59
Diarrhea	Index	3.48	0.276	-	2.93	4.03
	LCMF-ex	0.45	0.408	0.26964	-0.36	1.26
	LCMF-hy	-1.15	0.369	**0.00238**	-1.88	-0.42
	LFLF	-0.36	0.376	0.34653	-1.10	0.39
	LFHF	0.76	0.381	**0.04717**	0.01	1.52
Control	Index	3.08	0.496	-	2.09	4.08
	LCMF-ex	0.00	0.573	1.00000	-1.15	1.15
	LCMF-hy	-0.42	0.573	0.47041	-1.57	0.73
	LFLF	0.08	0.573	0.88491	-1.07	1.23
	LFHF	1.42	0.640	**0.03172**	0.13	2.70

Results fixed factors model for defecation frequency including diet, housing combination, gender, the simultaneous presence of food color, and interactions, with random factors for the individual animal and the order of diets. Both lower and upper confidence intervals are presented (Lower CL and Upper CL.) The index is diet STAN without the influence of food color or glitter. Significant P values are in bold.
