# Supplementary material for: Evaluation of commercial diets on fecal consistency and defecation frequency in rhesus macaques (Macaca mulatta) with chronic intermittent idiopathic diarrhea
Source: Lab Anim Res. 2025 May 20;41:15. doi: 10.1186/s42826-025-00246-6 (PMC12090390; doi:10.1186/s42826-025-00246-6)
Supplement: Supplementary file 4 — Additional file 4. [file 42826_2025_246_MOESM4_ESM.pdf]

Additional file 4

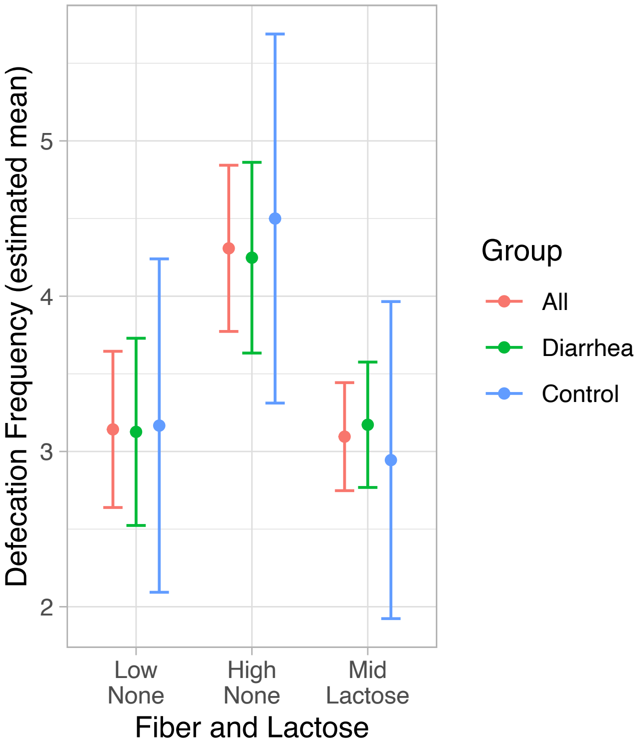

Figure A1 shows the estimated mean defecation frequency for all animals and for the diarrhea group and control group, the whiskers represent the 95% confidence interval. Fiber content is classified as Low, High, or Medium, and lactose as none when the diet was lactose-free. High fiber in the absence of lactose shows a clear increase in defecation frequency all groups compared to low fiber in the absence of lactose.
